# Barriers and facilitators to implementing parent-led infant pain care in rural settings: A qualitative descriptive study using the Theoretical Domains Framework and COM-B Model

**DOI:** 10.1080/24740527.2025.2602540

**Published:** 2026-02-05

**Authors:** Britney Benoit, Christine Cassidy, Jacqueline van Wijlen, Marsha Campbell-Yeo, Sionnach Hendra, Ruth Martin-Misener, Jennifer MacDougall, Ashley Cameron, Hannah McGee, Ripu Daman

**Affiliations:** a Rankin School of Nursing, St Francis Xavier University, Antigonish, Nova Scotia, Canada; b School of Nursing, Dalhousie University, Halifax, Nova Scotia, Canada; c Barrington Consulting Group, Halifax, Nova Scotia, Canada; d Public Health, Nova Scotia Health, Antigonish, Nova Scotia, Canada; e Women and Children’s Health Program, Nova Scotia Health, Antigonish, Nova Scotia, Canada; f Solutions for Kids in Pain, Halifax, Nova Scotia, Canada

**Keywords:** Breastfeeding, skin-to-skin contact, infant pain, implementation, qualitative, Theoretical Domains Framework, COM-B Model

## Abstract

**Aims:**

To support the implementation of parent-led infant pain care by identifying barriers and facilitators during acute procedures.

**Methods:**

We conducted a qualitative descriptive study guided by the Theoretical Domains Framework (TDF) and COM-B Model. We completed individual, virtual, semi-structured interviews with health system participants (hospital and community-based health care providers, clinical leaders, and administrators; *n* = 10) and parent participants (who had used hospital or community-based perinatal services in the last 12 months; *n* = 14) and analyzed the data using deductive-inductive qualitative content analysis.

**Results:**

Thirty-two themes were identified across the capability (9 themes), opportunity (13 themes), and motivation (10 themes) domains of the COM-B Model. Participants emphasized the influence of environmental context, resources, and social factors on the use of breastfeeding and skin-to-skin contact to manage infant pain across rural acute and community care settings. Health system and parent participants described similar barriers and facilitators, highlighting the inconsistent implementation of parent-led infant pain care.

**Conclusions:**

The barriers and facilitators identified in this study will inform the development of theory-informed, contextually relevant implementation interventions to support the use of best practice infant pain care in rural contexts.

## RÉSUMÉ

**Objectifs:** Soutenir la mise en œuvre d’une prise en charge parentale de la douleur chez le nourrisson, en cernant les obstacles et les facteurs facilitants lors de procédures aiguës.

**Méthodes:** Nous avons mené une étude descriptive qualitative reposant sur le cadre des domaines théoriques (CDT) et le modèle COM-B. Des entretiens individuels semi-structurés, réalisés virtuellement, ont été menés auprès d’intervenants du système de santé (prestataires de soins de santé en milieu hospitalier ou communautaire; responsables cliniques et administrateurs; n = 10) et de parents (ayant utilisé des services périnataux hospitaliers ou communautaires au cours des 12 derniers mois; n = 14). Les données ont ensuite été analysées à l’aide d’une analyse de contenu qualitative déductivo-inductive.

**Résultats:** Trente-deux thèmes ont été relevés dans les domaines de la capacité (9 thèmes), de l’opportunité (13 thèmes) et de la motivation (10 thèmes) du modèle COM-B. Les participants ont souligné l’influence du contexte environnemental, des ressources et des facteurs sociaux sur l’utilisation de l’allaitement maternel et du contact peau à peau pour la prise en charge de la douleur chez le nourrisson dans les milieux de soins aigus, en contexte rural et communautaire. Les intervenants du système de santé et les parents ont décrit des obstacles et des facteurs facilitants similaires, mettant en évidence la mise en œuvre non uniforme de la prise en charge parentale de la douleur chez le nourrisson.

**Conclusions**: Les obstacles et les facteurs facilitants mis en évidence dans cette étude éclaireront l’élaboration d’interventions de mise en œuvre fondées sur la théorie et adaptées au contexte afin de soutenir l’implantation de pratiques exemplaires de prise en charge de la douleur chez le nourrisson en milieu rural.

## Introduction

All infants experience procedural pain as part of routine care, such as intramuscular injections and heel lances, both in hospitals and in the community settings.^1–4^ When left untreated, pain can lead to adverse cardiorespiratory, hormonal, and neurodevelopmental outcomes in both preterm and full-term infants.^5–14^ In preterm infants, pain is associated with impeded tissue repair,^5^ poor body and head growth,^7^ reduced visual perceptual abilities,^14^ poorer language outcomes,^15^ and greater internalizing behaviors (such as anxiety and depression symptoms).^6,8^ In full-term infants, pain can cause structural and functional changes in the peripheral and central nervous system that are associated with increased sensitivity to pain during later procedures in infancy and childhood.^9–13^

Parent-led care, such as breastfeeding^a^^a^We are conscious of perpetuating oppressive and harmful discourses that do not reflect all families. Therefore, we used inclusive, gender-neutral language throughout data collection (including the use of terms such as “breast/chestfeeding” and “parent” to better reflect diverse lactation experiences).^16^ Gendered language of “breastfeeding” and “mother” are used throughout this article, as this is the language participants used to describe their experiences of supporting and providing parent-led infant pain care. and skin-to-skin contact, have evidence for pain-reducing efficacy and safety. In our systematic reviews of breastfeeding^17,18^ and skin-to-skin contact,^19^ we found that these interventions have the strongest evidence for reducing pain associated with acute tissue-breaking procedures. Direct breastfeeding was more effective than maternal holding, maternal skin-to-skin contact, topical anesthetics, and music therapy, and was as or more effective than sweet tasting solutions, such as oral sucrose, in full-term infants.^17^ Our review showed that skin-to-skin contact was more effective than no treatment, and as or more effective than sweet tasting solutions.^19^

Despite this evidence, parent-led infant pain care strategies are under-utilized in clinical practice. Although both nurses and parents describe positive perceptions regarding parent-led pain care, less than half of infants receive any pain-relieving intervention during tissue-breaking procedures.^20–22^ Most studies on pain management practices in infants have focused on the Neonatal Intensive Care Unit (NICU). Reasons for lack of uptake of parent-led pain care include parents’ and health care providers’ lack of knowledge,^23–25^ parental stress and anxiety,^23,25^ health care provider gatekeeping and exclusion of parents from providing pain care,^23–26^ and the restrictive nature of the care environments themselves.^23,24^ In contrast, facilitators of parent-led pain care include access to information and clear communication between parents and providers,^23,27^ parental motivation to be active participants in their infants’ care,^23–25,27^ and family-friendly physical environments.^23,24^

Few studies have explored how to support the uptake of parent-led pain care outside of neonatal units. Most infants undergo painful procedures as part of healthy infant care delivered by postpartum clinical services, primary care providers, and community public health offices. Implementing evidence-informed practices successfully requires a comprehensive understanding of the barriers and facilitators to change, as well as tailoring implementation interventions to the local context.^28^ To date, a theoretically informed approach to identify behavioral determinants of change and the implementation interventions needed to support parent-led pain care in rural acute and community care contexts has not been described. To guide this study, we used the COM-B^29^ model and the Theoretical Domains Framework (TDF)^30,31^ to identify behavioral determinants that influence the uptake of parent-led infant pain care. The aim of this study was to identify evidence of barriers to, and facilitators of, parent-led pain care in infants during routine acute procedures.

## Materials & methods

### Theoretical framework

This article reports on Phase 1 of a two-phased study described in a previously published protocol.^32^ Phase 1 followed a systematic, theoretically informed approached guided by the COM-B Model^29^ and the Theoretical Domains Framework (TDF).^30,31^ The COM-B Model describes that behavior arises through the interaction of an individual’s capability (psychological and physical skills), opportunity (physical and social environments), and motivation (automatic and reflective processes), offering a simple yet comprehensive framework to understand behavior change in health care settings. The TDF is an integrated framework that expands on the components of the COM-B model by breaking down the determinants of behavior into 14 domains (e.g., knowledge, skills, beliefs about capabilities, environmental context and resources, social influences; Supplemental Table 1) offering a detailed and practical guide for identifying barriers and facilitators to implementation. Both frameworks were developed through expert consensus and theoretical synthesis to integrate multiple behavior change theories into practical tools for implementation research.^29,30^ We selected these frameworks because they are widely validated and used in implementation science to provide a shared language and structure for understanding complex behaviors, identifying barriers and facilitators to evidence use, and designing interventions for health care contexts.^29–31,33,34^ This aligns directly with our aim to identify determinants of parent-led pain care to inform future implementation strategies for rural hospital and community settings.

### Design

We utilized a qualitative descriptive design and conducted individual interviews to capture participants experiences in their own words and generate context-specific insights.^35,36^ This approach supported systematic identification of barriers and facilitators to parent-led pain care while maintaining fidelity to participant perspectives.^35^ Individual interviews allowed for in-depth exploration of participants lived experiences, promoted a safe environment for participants to discuss potentially sensitive experiences with parent-led infant pain care, and ensured diverse perspectives across health system and parent participants were represented.^37^

### Setting and sample

This study was conducted in regional hospitals and community-based contexts that provide perinatal and infant care in the northeastern region of the Atlantic Canadian province of Nova Scotia. Nova Scotia is made up of a mix of urban and rural communities, with nearly half of the population living in rural areas.^38^ Two health service organizations deliver perinatal and infant care in the province: IWK Health and Nova Scotia Health. IWK Health, located in the capital city of Halifax, is the province’s only tertiary perinatal referral center and supports approximately 4,500 births annually.^39^ Nova Scotia Health provides pregnancy, birth, and postpartum care for approximately 3,000 births annually in community hospitals and public health offices across the entire province with four service zones: Northern, Eastern, Central, and Western.^40^ In the Eastern Zone, perinatal and infant services are primarily delivered from St. Martha’s Regional Hospital, which provides regional maternity services including prenatal, birth, and postpartum care, and Cape Breton Regional Hospital, which includes a NICU. Community-based primary care providers administer most infant immunizations, with public health nurses supporting infants without a primary care provider. We completed stratified, purposive sampling^30,41^ to recruit both hospital and community-based health care providers (e.g., acute care nurses, acute and primary care physicians, family practice nurses, laboratory technicians, midwives, public health nurses), clinical leaders (e.g., lactation consultants), administrators (e.g., program managers), and parents who had used hospital or community perinatal services in northeastern Nova Scotia in the last 12 months. To recruit health system participants, our research team circulated a recruitment e-mail containing study details and an invitation to participate through key research team partners and networks. For parent participant recruitment, we shared electronic posters on social media and placed printed copies in hospital and community antenatal care areas.

### Data collection

We received ethical approval for this study from the Nova Scotia Health Research Ethics Board in December 2020 (approval # 1026216). Potential participants were sent an electronic copy of the study information and consent form. The principal investigator or research coordinator then conducted a virtual informed consent discussion, reviewing the study purpose, processes, potential risks and benefits, and the voluntary nature of participation. Participants were encouraged to ask questions prior to providing consent. Verbal consent was documented and each participant received a completed copy of the consent form.

The principal investigator or research coordinator conducted an individual, semi-structured, virtual interview with each consenting participant. The principal investigator has over 10 years of qualitative research experience in perinatal implementation studies and the research coordinator received focused training and mentorship in qualitative interviewing best practices. The semi-structured interview guide for parent (Table 1) and health system participants (Table 2) were developed using the TDF domains^30,31^ (Supplemental Table 1) to ensure coverage of relevant behavioral determinants. During the interview, we used follow-up probes that were responsive to participant language and direction, ensuring that data collection was grounded in participant experiences.^37^ We pilot tested the interview guides with both a parent partner and health system partner to ensure the interview questions were appropriate and did not constrain participant responses, and that the interview was feasible to complete within a 60- to 90-min timeframe. We had no prior relationships with participants prior to study enrollment. To support reflexivity regarding the researcher-participant relationship during data collection, we documented field notes, engaged in ongoing self-reflection on personal assumptions and professional experiences, and debriefed after each interview.^37,42,43^

**Table 1.** Semi-structured interview guide (parent participants).

Topics	Questions
Knowledge	Have you or others you know used breast/chestfeeding and/or skin-to-skin contact to manage your babies’ pain? Tell me a little bit about that experience/what you know about using breast/chestfeeding or skin-to-skin contact to manage your baby’s pain.How do/did you find information about using breast/chestfeeding or skin-to-skin contact for managing your baby’s pain?
Skill	What knowledge or supports do you use to breastfeed or provide skin-to-skin contact to your baby during pain? Are there additional knowledge or supports that you need to breastfeed or provide skin-to-skin contact to your baby during pain?
Intentions and goals	How important do you feel it is for your baby to be breast/chestfed or be in held skin-to-skin contact during pain?
Beliefs about consequences	Are there any benefits to using breast/chestfeeding or skin-to-skin contact to manage your baby’s pain? Are there any negatives to using breast/chestfeeding or skin-to-skin contact to manage your baby’s pain?
Environmental context and resources	What factors influence you using skin-to-skin contact or breast/chestfeeding to manage your baby’s pain? (Prompt(s): Stressors, resources, barriers, facilitators).
Beliefs about capabilities	How confident do you feel in your ability to breastfeed or provide skin-to-skin contact to manage your baby’s pain? (Prompt: Is there anything that would make you more confident?)Are there challenges related to breast/chestfeeding or providing skin-to-skin contact for your baby when they are in pain? (Prompt(s): Is there anything that would make using breast/chestfeeding/skin-to-skin contact for your baby during pain easier?)
Social influences	Do your family/friends influence your decision to use breast/chestfeeding or skin-to-skin contact to manage your baby’s pain? (Prompt(s): If yes, how would they influence your decision? To what extent do they influence your decision?)
Emotion	Do emotions, both positive or negative, influence your decision to use skin-to-skin contact or breast/chestfeeding for your baby’s pain management? (Prompt(s): Fear of consequences of using/not using, anxiety, stress).
Conclusion	Are there any other key things related to using breast/chestfeeding or skin-to-skin contact to manage your baby’s pain that were not discussed today that you think are important to talk about?

Note. Based on recommendations from the American Academy of Breastfeeding Medicine [1], the language breast/chestfeeding and parent was used in study documents to reflect diverse lactation experiences.

**Table 2.** Semi-structured interview guide (health system participants).

Topics	Questions
Knowledge	Have you or others you know used breast/chestfeeding and/or skin-to-skin contact to manage infant pain? Tell me a little bit about that experience/what you know about using breast/chestfeeding or skin-to-skin contact to manage infant pain.How do/did you find information about using breast/chestfeeding or skin-to-skin contact to manage infant pain?
Skill	What knowledge, resources, or skills do you use to support breast/chestfeeding and/or skin-to-skin contact to manage infant pain? Are there additional knowledge, resources, or skills that you need to support breast/chestfeeding and/or skin-to-skin contact to manage newborn pain?
Intentions and goals	How important do you think it is for infants to have breast/chestfeeding or be held in skin-to-skin contact for pain management during procedures? If important, what actions have you taken toward using these strategies for pain management?
Beliefs about consequences	Are there any benefits to using breast/chestfeeding or skin-to-skin contact to manage infant pain? Are there any negatives or harms to using breast/chestfeeding or skin-to-skin contact to manage infant pain?
Environmental context and resources	What factors influence your decision or ability to use skin-to-skin contact or breast/chestfeeding for infant pain management? (Prompt(s): Stressors, resources, organizational culture, barriers, facilitators).
Beliefs about capabilities	How confident do you feel in your ability to support breast/chestfeeding or skin-to-skin contact to manage infant pain? (Prompt: Is there anything that would make you more confident?)Are there challenges related to supporting breast/chestfeeding or skin-to-skin contact for infants during painful procedures? (Prompt(s): Is there anything that would make supporting breast/chestfeeding/skin-to-skin contact for infants during pain easier?)
Social/professional role identity	Do you feel like you have a responsibility to use pain management strategies for infants? Why or why not? Have you or others you know acted as a leader to support breast/chestfeeding and/or skin-to-skin contact for infant pain management? (Prompt: If yes, what does that leadership look like in your organization and/or experience?)
Social influences	How do your colleagues influence your decision to support breast/chestfeeding or skin-to-skin contact to manage infant pain? (Prompt(s): To what extent do they influence your decision?)
Reinforcement	Are there any incentives for you to support skin-to-skin contact or breast/chestfeeding for infant pain management?
Emotion	Do emotions influence your decision to support skin-to-skin contact or breast/chestfeeding for infant pain management? (Prompt(s): Fear of consequences of using/not using, anxiety, stress, burnout?)
Conclusion	Are there any other key things related to supporting breast/chestfeeding or skin-to-skin contact to manage infant pain that were not discussed today that you think are important to talk about?

Note. Based on recommendations from the American Academy of Breastfeeding Medicine [1], the language breast/chestfeeding and parent was used in study documents to reflect diverse lactation experiences.

### Data analysis

We summarized participant demographic characteristics using descriptive statistics. Interview transcripts were analyzed using a combined deductive-inductive qualitative content analysis approach,^42,44,45^ informed by established practices in implementation science research.^46^ We used NVivo (QSR International)^47^ software to support data management and coding.

#### Step 1 – deductive coding

First, two reviewers (BB, RD) used directed content analysis to deductively categorize participant interview responses into the 14 domains of the TDF.^44,45^ This step facilitated structured organization of data into theoretically relevant domains.

#### Step 2 – inductive coding

Second, within each of TDF domain, we applied inductive (conventional) qualitative content analysis^44^ to identify themes of barriers and facilitators grounded in the participants words and experiences. To do this, we read interview transcripts line-by-line multiple times to generate initial codes and then synthesized those codes into higher level themes of relevant barriers and facilitators.^35,37^ Thematic saturation guided sample size throughout iterative data collection and analysis. We considered themes saturated when no new codes or themes were identified in successive interviews and when existing themes were well-developed.^48,49^ Consistent with thematic analysis recommendations, we did not quantify the frequency of individual responses. Themes were identified based on their conceptual significance and relevance to the research question, rather than numerical prevalence.^50^ During analysis we completed regular consensus meetings to review coding, resolve discrepancies, and refine emerging themes. This two-step approach ensured theoretical grounding and responsiveness to participants unique perspectives.

#### Trustworthiness

We followed established strategies to promote credibility, transferability, dependability, and confirmability.^43,51,52^ These included clear documentation of the research process, including sampling, participant characteristics, and the health system context; maintaining an audit trail of coding and theme development; reflexive journalling to acknowledge positionality (including the lead researcher’s background as a Registered Nurse with over 10 years of experience researching and supporting breastfeeding and skin-to-skin contact for infant pain management); and regular team debriefings to discuss positionality and promote analytic rigor.

## Results

A total of 24 participants completed a study interview (*n* = 10 health system participants, defined as health care providers, clinical leaders, and health administrators; *n* = 14 parent participants who accessed hospital or community perinatal services in the previous 12 months). Table 3 summarizes participant demographic information. We analyzed participant responses regarding barriers to, and facilitators of, parent-led infant pain care across health care contexts. We present the identified barriers and facilitators within each of the three domains of the COM-B model: capability (Table 4), opportunity (Table 5), and motivation (Table 6). Within each domain, participant responses highlight specific barriers and facilitators mapped onto the corresponding TDF domains, illustrating how the two frameworks were applied in the analysis.

**Table 3.** Health system and parent participant demographics.

	Health Care Provider Participants		NICU Parent Participants		General Parent Participants	
	N (%)	M (range)	N (%)	M (range)	N (%)	M (range)
Gender identity Woman	10 (100)	–	5 (100)	–	9 (100)	–
Ethnicity White First Nations	10 (100) 0 (0)	–	5 (100) 0 (0)	– –	9 (100) 1 (11)	– –
Occupation Nursing Allied health professional Physician Administrative role Non-health care role	6 (60) 2 (20) 1 (10) 0 (0) 1 (10)	– – – – –	2 (40) 1 (20) 0 (0) 1 (20) 1 (20)	– – – – –	4 (44) 3 (33) 0 (0) 2 (22) 1 (11)	– – – – –
Educational background Diploma Bachelor’s degree Master’s degree Doctorate degree	2 (20) 6 (60) 2 (20) 0 (0)	– – – –	3 (60) 2 (40) 0 (0) 0 (0)	– – – –	2 (22) 4 (44) 2 (22) 1 (11)	– – – –
Approximate number of years working with infants	–	14.15 (2–35)	N/A	N/A	N/A	N/A
Approximate number of years working in rural Nova Scotia	–	14.5 (1.5–35)	N/A	N/A	N/A	N/A
Number of hours worked (weekly) Full-time Part-time	8 (80) 2 (20)	–	N/A	N/A	N/A	N/A
Number of children currently under 12 months of age	N/A	N/A	–	1 (1–3)	–	1 (1)
Total number of children	N/A	N/A	–	2 (1–4)	–	2 (1–3)
Approximate number of years accessing health care in rural Nova Scotia	N/A	N/A	–	21.8 (0–37)	–	19.3 (7–33)
Approximate duration (days) of most recent stay in the CBRH NICU	N/A	N/A	–	47 (13–78)	–	N/A

Notes. N = 24. Total number of children and number of children currently under 12 months of age reported in median and corresponding range.

**Table 4.** Themes under capability domains of COM-B Model.

COM-B	TDF Domains	Barriers and Facilitators	HS	P
Psychological capability	Knowledge	Health care provider education	**✓**	**✓**
		Parent education	**✓**	**✓**
	Cognitive and interpersonal skills	Relational care	**✓**	
		Life experience and growth over time	**✓**	**✓**
	Memory, attention and decision processes	Memory of education	**✓**	
		Focused practice	**✓**	
Physical capability	Physical skills	Skills in breastfeeding, latching, SSC	**✓**	**✓**
		Disciplinary differences	**✓**	**✓**
		New set of physical skills	**✓**	

Note. HS represents health system participants and P represents parent participants. A checkmark (✓) indicates that the corresponding barrier or facilitator was described by participants from that group, and that thematic saturation was achieved within that group for the given barrier or facilitator (i.e., no new relevant information emerged in successive interviews).

**Table 5.** Themes under opportunity domain of COM-B Model.

COM-B	TDF Domains	Barriers and Facilitators	HS	P
Opportunity	Environmental and context resources	NICU & nursery environment	**✓**	**✓**
		OR & cesarean births	**✓**	**✓**
		Laboratory staff & in-patient settings	**✓**	**✓**
		Midwifery care	**✓**	**✓**
		Lactation consultant care	**✓**	**✓**
		Physical environment & time in primary care	**✓**	**✓**
		Unique challenges in rural maternal-child care	**✓**	
	Social influences	Leadership & interprofessional collaboration	**✓**	
		Champions & peer role modeling	**✓**	
		Generational differences in practice	**✓**	
		Position & influence of health care providers	**✓**	**✓**
		Social acceptance of breastfeeding		**✓**
		Peer & family influence on breastfeeding		**✓**

Note. HS represents health system participants and P represents parent participants. A checkmark (✓) indicates that the corresponding barrier or facilitator was described by participants from that group, and that thematic saturation was achieved within that group for the given barrier or facilitator (i.e., no new relevant information emerged in successive interviews).

**Table 6.** Themes under motivation domain of COM-B Model.

COM-B	TDF Domains	Barriers and Facilitators	HS	P
Motivation	Reinforcement	Seeing the benefits	**✓**	**✓**
		Positive feedback to colleagues	**✓**	
	Emotions	Negative & positive emotions	**✓**	**✓**
	Beliefs about capabilities	Varied confidence across providers	**✓**	**✓**
		Increased parent confidence	**✓**	**✓**
	Beliefs about consequences	Benefits to infant & mother	**✓**	**✓**
		Benefits to health care providers	**✓**	
		Potential for negative outcomes	**✓**	**✓**
	Social and professional role identity	Professional role of the Registered Nurse	**✓**	
		Parenting role to provide comfort	**✓**	**✓**

Note. HS represents health system participants and P represents parent participants. A checkmark (✓) indicates that the corresponding barrier or facilitator was described by participants from that group, and that thematic saturation was achieved within that group for the given barrier or facilitator (i.e., no new relevant information emerged in successive interviews).

### Capability

The following results describe participant perspectives regarding barriers and facilitators to capability, organized by the relevant TDF domains. These include components of psychological capability and physical capability. Table 4 summarizes all barriers and facilitators to capability identified by participants.

#### Psychological capability

Participants emphasized how their psychological capability directly impacted their ability to utilize parent-led infant pain care. Both parents and health system participants pointed to knowledge as a facilitator of using breastfeeding and skin-to-skin care for pain. Health system participants stressed the importance of consistent, ongoing education on parent-led pain care for all providers so that they can support breastfeeding and skin-to-skin care during procedures. Health system participants recommended a diversity of education strategies to ensure this knowledge was acquired, including clinical education days, videos, regular team meetings, practice newsletters, and one-on-one education and mentorship with lactation consultants. All participants identified prenatal parent education as a strategy to prepare parents to be advocates for their newborns pain care. As Health System Participant 05 noted, the parents who receive education “are requesting it. … Then it’s like if they’re saying they want to do skin-to-skin, then how do you refuse that?” Parent participants reported that this education was currently being delivered by nurses, midwives, and lactation consultants, whereas it was less consistently being provided by physicians and laboratory staff.

Participants also described how cognitive and interpersonal skills, especially relational care and experience over time, facilitated effective parent-led pain care support. Health system participants emphasized the value of relational practice when supporting breastfeeding and skin-to-skin for pain care, such as being patient, encouraging, and letting the breastfeeding parent take the lead. As one health system participant described:

Just allowing that space so being patient and supportive are two really big things that I think anybody can do and normalizing newborn behaviors. And encouraging, that’s another really big part, I think. Encouraging the mom to take their time to do what they need to do. (Health System Participant 01)

Health system participants noted that, with time and experience, they grew more confident supporting breastfeeding and skin-to-skin, fostering a sense of comfort in providing this care. Similarly, parent participants shared that having experience with a previous child made them more confident in providing and advocating for parent-led pain care for their younger children. When discussing the role of memory, attention, and decision processes, health system participants described the benefit of having a dedicated role and focused practice, such as a being an International Board Certified Lactation Consultant (hereafter referred to as lactation consultant). One lactation consultant participant described how this helped them place attention on parent-led pain care:

Breastfeeding is my speciality. So that’s always my focus and I can always focus on that. Whereas a staff nurse on the unit has a million other things or priorities of focus that she’s focusing on, as opposed to the breastfeeding that is one piece of her head, where mine is always in that mode. And I also have the time; you know maybe I have more time to focus on those conversations with the parents to empower them. (Health System Participant 02)

#### Physical capability

Both health system and parent participants emphasized that physical skills play a key role in supporting parent-led pain care. Comfort with specific skills, such as latching a baby to the breast, facilitating/providing skin-to-skin, hand expression of breast milk, and handling newborns were described as necessary for these practices. For example, one health system participant explained:

One of the things I think has been helpful, especially for any bloodwork or injections that are done early is being able to assist with breastfeeding […] or at least kind of allowing for the environment and space for people to feel comfortable to get baby latched. (Health System Participant 01).

Health system participants described disciplinary differences in these physical skills. For example, lactation consultants were seen as having these skills, whereas laboratory staff were perceived as being less comfortable with handling newborns and supporting breastfeeding and skin-to-skin. Health system participants noted that parent-led pain care often required a new set of physical skills and body positioning that differed from completing procedures in an incubator or infant cot. Parent participants stressed the importance of health care staff taking the time for hands-on teaching with parents about infant positioning for procedures that is responsive to parent comfort.

### Opportunity

Participants described how both physical and social opportunity influenced their ability to engage in parent-led infant pain care. Table 5 summarizes participant-identified barriers and facilitators within this COM-B domain.

#### Physical opportunity

Participants described how the environmental context and resources shaped their opportunity to provide parent-led infant pain care (Table 5). The NICU and nursery context, cesarean birth in the operating room, and laboratory staff on in-patient units were frequently described as presenting barriers. Both parent and health system participants noted that critical infant health status, fear of moving infants from their incubator, and open-concept NICU and nursery environments created separation and lack of parental contact during procedures. Cesarean births in the operating room were described as presenting unique challenges to parent-led pain care. Health system participants specifically noted that limited physical space due to the surgical field and monitoring equipment often prevented immediate skin-to-skin on the birthing parent’s chest. In rural settings, post-operative recovery rooms were described as busy with competing demands, diverse patient needs, and lack of nursing staff to monitor both the birth parent and infant in skin-to-skin care. Both health care providers and parents described separation immediately following cesarean birth as common practice. One parent shared that she was unaware of whether her newborn has undergone any painful procedures during the time they were apart:

“I would note, as well, that he was in recovery for about 45 minutes without me because I had a C-section. So my partner was with him, and I was not. So I don’t really know what took place in those 45 minutes” (Parent Participant 08).

Parents indicated that laboratory staff on in-patient units inconsistently offered parent-led pain care. Health system participants attributed this variability to time constraints, lack of collaboration across providers, and lack of resources in the physical environment. Participants noted that laboratory staff often lacked the time or physical set-up to facilitate skin-to-skin or breastfeeding prior to procedures. Ensuring ergonomic safety and comfort during blood draws, such as having portable stools and space beside the patient bed or chair, were seen as necessary to support this practice.

In contrast, midwifery and lactation consultant care were described as key facilitators of parent-led pain care. Long midwifery prenatal appointments and postpartum home visits offered integrated, family-centered assessments and care where parent-led pain care was explained and supported. Both parents and health system participants emphasized that completing blood work in families’ homes created a more comfortable environment to breastfeed during procedures. Access to a lactation consultant run clinic that provided prenatal education and postnatal breastfeeding support was also described as an ideal setting for infant procedures. This clinic setting offered access to lactation consultant support with breastfeeding; collaboration between lactation consultants, laboratory staff, and families; appropriate physical infrastructure (e.g., ergonomic stool for staff, comfortable reclining chair for families); and sufficient time to facilitate parent-infant contact:

“But really in our clinic, there’s no option to not do it. That we set up … the stool gets set up. Like, everything is set up. We warm … the baby’s foot is warmed up. We have the mom breastfeeding. There’s no option not to do it. Like, it’s going to be done” (Health System Participant 04).

Both health system and parent participants identified specific barriers to parent-led pain care in primary care settings. Parents noted that lack of clinic space and short appointment times often made it difficult to breastfeed and provide skin-to-skin for immunizations in family physician offices. A common suggestion was the need for a dedicated, private vaccination room with a comfortable recliner chair where one could breastfeed before, during, and after the procedure, without feeling rushed to leave so that the next patient could be seen. Longer appointment times for infant immunizations was also suggested as a facilitator of using these strategies effectively.

Health system participants described the unique challenges faced within rural children and women’s hospital units. These included nursing staffing shortages and large numbers of off-service patients on perinatal units. Participants emphasized that the number of Registered Nurses staffing rural units was insufficient to meet the complex care demands across labor and birth, OR, recovery, and post-partum care for perinatal populations. Frequent staff turnover, many new staff, and staff redeployment to other practice areas to fill deficits limited their capacity to provide optimal, evidence-informed care to infants and their families. The increasing presence of high acuity off-service patients with complex care needs on rural perinatal units was seen as particularly disruptive. Participants explained that these patients drew focus away from infant and maternity care, making it harder to prioritize or support breastfeeding and skin-to-skin. One participant noted:

It’s not an obstetrical unit here anymore. So unlike [specialty hospital] where you could just have a postpartum unit, and people just totally designated to do […] Getting the same messages, caring for the same clientele, doing the same things day after day, the reality of that just isn’t here. […] So the health of mom and babe might be their last priority. And that’s unfortunate, but it’s the reality. (Health System Participant 04)

#### Social opportunity

Participants described social influences (Table 5) on parent-led pain care. Health system participants specifically spoke to the role of leadership and interprofessional collaboration, champions and peer role modeling, and generational differences shaping clinical practice. Collaboration within and across disciplines was seen as enabling practice uptake. For example, participants suggested that better communication and collaboration between laboratory and nursing staff around positioning infants for procedures could facilitate greater use of breastfeeding and skin-to-skin care. Senior leadership buy-in was identified as esential for supporting best-practice in maternal–child health. However, participants noted loss of dedicated senior leaders for maternity and pediatric services with “power and authority” within rural settings contributed to de-prioritization of maternal-infant health issues within the system, limited resource availability, reduced communication and validation of practice efforts, and delays in evidence-informed practice changes. As one participant described:

We don’t really have a maternity and pediatrics services; we just have a chief medical officer for the zone. […] I feel like it’s not going to be at the top of their list. […] It could still be years before we get anything. (Health System Participant 03)

The presence of pain care champions was seen as a strategy to facilitate parent-led pain care. Peer-to-peer influence, particularly from colleagues who demonstrated a commitment to the practice, encouraged uptake within units and across organizations. Participants also described generational differences in clinical practices. Parent-led care was often not the norm for more senior staff, however, peer modeling was seen as an effective strategy to encourage cultural shift and increased uptake of evidence-informed care.

The social position and influence of health care providers greatly shaped whether parent-led pain care occurred. Parent participants highlighted that health care providers hold a position of power and authority to either facilitate parent-led pain care or prevent its use. Some parent participants shared that they advocated for the use of parent-led care when a health care provider did not encourage or support it; however, others spoke about feeling they “have to be a good patient” and not request it. For example, one parent described:

Feeling the pressures of time constraints when taking my baby to have like, immunizations, or something like that […] I think when I’m going to these appointments; I feel the kind of pressure to be a good patient and like, to go along with the pace and the direction that our physicians set. (Parent Participant 16)

Parent participants also noted that social acceptance of breastfeeding greatly influenced their comfort using it for infant pain management. For example, parent participants described generational and gendered differences in breastfeeding acceptance and emphasized the need to normalize public breastfeeding. Support from peers and family members was seen as essential for both breastfeeding success and its use as a pain management strategy. As one parent noted:

When it came to this consoling an upset baby or […] whether it be at home or in the doctor’s office, yeah, it did help. But as I mentioned, there were a few times that I just thought I don’t want to go nurse in this space. And I think it came down to not how comfortable was the space in terms of, you know, resources, but who was in the room. (Parent Participant 06)

### Motivation

The following section describes barriers and facilitators related to motivation, organized by the relevant TDF domains. Table 6 summarizes the barriers and facilitators identified by participants for this domain.

Participants described numerous factors that positively reinforced the use of parent-led pain care. Both parents and health system participants noted that “knowing the benefits” and “seeing it firsthand” (Health System Participant 05), such as reduced infant pain, increased parent control and confidence, and easier blood collection encouraged them to use these practices. Health system participants also described positive feedback to and from colleagues and leadership, such as encouraging and saying “you guys are doing a good job” (Health System Participant 02) when parent-led care was used, as a key form of positive reinforcement. Parent participants noted factors than negatively reinforced the use of breastfeeding, including the burden and workload for the breastfeeding parent and the inconvenience of having to unlatch their baby immediately after an immunization while the baby was still actively feeding.

Parents and health system participants both described strong, yet unique, emotions associated with use of parent-led care. Health system participants acknowledged experiencing stress and frustration when supporting parent-led care in their resource-limited work environments but also described feeling pride in their practice and the positive emotions when empowering parents to be active participants in care. Parent participants reflected on the anxiety, guilt, and exhaustion of caring for a newborn, yet emphasized that the comfort, closeness, calm, and satisfaction of comforting their baby during procedures helped motivate them to breastfeed and provide skin-to-skin:

I feel like holding them and breastfeeding them, it just makes me more comfortable. I don’t have to hear them cry for an extended period of time. […] Having that comfort for them of having the skin-to-skin and having the breastfeeding just to kind of be like okay, ow, that hurt but, oh, mom’s there, and here’s my comfort […] being able to comfort them in some way just helps my anxiety, and theirs. (Parent Participant 07)

Health system participants expressed varied belief in their capabilities to support parent-led pain care. Midwives and lactation consultants reported feeling the most confident in supporting breastfeeding and skin-to-skin, while laboratory staff were perceived as being less confident in handling newborns and supporting parental involvement during procedures. Both parent and health system participants agreed that providing pain care helps make parents more confident in their ability to care for and comfort their baby. Participants described numerous positive consequences for infants and parents. These included less pain, faster recovery, improved physiologic stability, and the prevention of long-term negative health outcomes for infants and empowerment, self-efficacy, confidence, and improved mental health for mothers. For example, Parent Participant 06 described: “I could almost tell that it did make a difference immediately. I mean, she just was happier and more calm. […] I noticed a big difference in her distress.” Health system participants described contrasting perspectives on ease of using parent-led pain care: some noted that it could make procedures easier; others felt that it makes some procedures (like intra-venous line starts) more challenging. Participants across groups agreed that comforting infants during procedures is the parents’ role. Registered Nurse participants emphasized their professional responsibility to promote and support parent-led pain care and “meet the gold standard” (Health System Participant 03) as a “moral responsibility” (Health System Participant 15) within their practice.

## Discussion

This study is the first to use the Theoretical Domains Framework and COM-B Model to systematically identify the barriers and facilitators to parent-led infant pain care in rural community and hospital contexts. Both parents and health system participants described how capability, opportunity, and motivation influence the involvement of parents as active participants in provision of pain care to their infants during acute procedures. Overall, health system and parent participants offered closely aligned perspectives.

Our findings suggest that the environmental context plays an instrumental role in either supporting or hindering the use of breastfeeding and skin-to-skin contact to manage pain in rural acute and primary health care settings. Participants on our study reiterated challenges to implementing skin-to-skin contact and breastfeeding in the operating room and NICU contexts that have been previously reported in the literature. The restrictive medicalized environment, with equipment and technology, lack of physical space for parents, inadequate staffing, and polices that limit parental involvement creates separation and contributes to the underutilization of parent-led pain care.^23,24^ Participants in our study also identified unique barriers to supporting breastfeeding and skin-to-skin contact during painful procedures in rural regional hospital maternal–child units. In these units, staff often provide care to families across labor and birth, postpartum, pediatrics, and women’s health specialties. High health system demand has led to an increased number off-service patients with complex medical needs being placed on maternal–child units. When combined with inadequate staffing, participants described an environment in which perinatal care becomes deprioritized and skin-to-skin and breastfeeding (both during and outside of painful procedures) receive inadequate support. A recent qualitative descriptive study conducted at 10 rural hospital sites in British Columbia, Canada identified similar barriers to providing equitable, evidence-informed maternity care.^53^ That study highlighted challenges in maintaining knowledge and skills in obstetrical care, recruitment and retention of maternity nurses and physicians, and financial instability as barriers to quality care. Amidst these ongoing pressures on the healthcare system, health care providers and administrators must not overlook the importance of low-tech and person-centered interventions such as parent-led infant pain care.

Participants in our study emphasized the need for invested leadership, at local and provincial levels, who can allocate adequate resources to prioritize maternal–child care. Participants viewed such leadership as capable of influencing providers’ capability, opportunity, and motivation to implement parent-led pain care by supporting regular education and skills practice, advocating for adequate staffing and focused perinatal practice environments, acting as champions and role models for parent-led care practice, and providing positive reinforcement and feedback to staff who implement parent-led pain care. Canada’s National Pediatric Pain Management Standard underscores the role of organizational leadership in supporting equitable, evidence-informed pain care.^54^ This standard calls on leaders to establish a pediatric pain management framework for their organizations and to dedicate resources to improving pediatric pain management practices. Recommended key resources include an interdisciplinary pain committee dedicated to pediatric pain management, organizationwide policies specific to pediatric pain assessment and management, and organizationwide policies that promote person- and family-centered approaches to pediatric pain care delivery. These recommendations align with our participants’ perspectives that effective implementation of parent-led pain care requires interdisciplinary leadership, integrated care, and a family-centered care culture across providers, practice environments, and organizations.

Parent and health system participants specifically highlighted barriers to breastfeeding during immunization appointments in primary care practices. These barriers included lack of time; feeling rushed during appointments; lack of clinic space to breastfeed before, during, and after the immunization; and the absence of explicit recommendation or support from primary care providers to breastfeed during the procedure. Participants recommended dedicating a separate space in primary care offices for infant immunizations, as breastfeeding parents could continue feeding their baby after the immunization was complete, rather than having to unlatch or move to a public waiting room. Parent participants repeatedly emphasized that provider support of breastfeeding strongly influenced their use of breastfeeding for infant pain management in community settings. Although some parents actively advocated to breastfeed during procedures, others noted that they would breastfeed only if their primary care provider explicitly recommended it. This finding is consistent with studies conducted in the NICU context, where communication about the parent role during procedures, practical guidance on how to hold their baby, and active collaboration between the health care provider and parent facilitated parent-led pain care.^24,26^

Lack of general social acceptance was also repeatedly raised, with parents noting that in small, rural communities, they did not always feel comfortable breastfeeding in public spaces – which negatively impacted their comfort with breastfeeding during procedures. Previous research has identified social acceptance as a key determinant of breastfeeding,^55^ with negative perceptions of public breastfeeding being associated with concealing breastfeeding^56^ and shorter breastfeeding durations.^57^ Chan and Whitfield^58^ recently explored perceptions of breastfeeding in public spaces using photo elicitation methods in a socio-economically diverse sample of participants in Nova Scotia, Canada. Their findings showed that women who were parents reported higher comfort scores in response to photos of infants being breastfed in public compared to men and non-parents. Parent participants in our study emphasized the importance of social acceptance of breastfeeding within both public and clinical spaces to enable their confidence breastfeeding. Parents also highlighted the crucial role of the patient–provider relationship in making breastfeeding during procedures a comfortable experience for them.

## Strengths & limitations

This study has several limitations. First, the sample was relatively homogenous, with most having a high level of education and identifying as White women. Despite purposive sampling efforts, we were unable to capture the experiences of sex- and gender-diverse participants, participants from lower socio-economic backgrounds, and those from racially and culturally diverse communities. As such, fundings may not reflect the experiences of these populations. Although health system participants represented a range of roles, most were nurses. Despite repeated efforts to recruit physicians and laboratory staff, their representation was limited. This may have limited the diversity of interprofessional perspectives on implementation of parent-led pain care. Although the TDF and the COM-B model provided a structure for exploring barriers and facilitators to implementation, there is a risk that using this approach unintentionally constrained participant responses. Strengths of this study include recruitment from across acute care, primary care, and public health settings, which enhanced representation of the contexts in which infant pain care is provided. The use of the TDF and COM-B model provided a rigorous, theory-informed approach to data collection and analysis, enabling a comprehensive exploration of the complex factors influencing implementation of breastfeeding and skin-to-skin care for procedural pain management.

## Directions for future research

Taken together, this study highlights numerous barriers and facilitators to parent-led pain care for infants during routine procedures in rural acute and community health care contexts. Our findings underscore the important influence of environmental context, resources, and social factors on the use of breastfeeding and skin-to-skin contact to manage infant pain. Building on these findings, future research should focus on co-designing, implementing, and evaluating targeted, theory-informed interventions that address the identified barriers and leverage facilitators of parent-led pain care in rural settings. Using implementation science frameworks such as the COM-B model, our research team plans to collaborate with parents, health care providers, and administrators to develop tailored strategies for the unique environmental, organizational, and social realities of rural acute and community care. These strategies may include targeted parent and provider education, leadership engagement, workflow adjustments, changes to the physical environment, and the promotion of parent-led pain care champions to support practice change. There is a critical need for research that assesses implementation outcomes, such as reach, acceptability, cost, and sustainability of interventions to support parent-led infant pain care across diverse rural health care settings.^59,60^ Embedding and evaluating interventions within local quality improvement initiatives may offer an effective approach to support practice change, foster ownership among health care teams, and promote sustainable integration into care.^61–63^ Additional research in this area can contribute to context-specific and scalable strategies to advance parent-integrated, effective pain management for infants during acute procedures.

## Supplementary Material

Supplemental Material
