# Supplementary material for: Barriers and facilitators to implementing parent-led infant pain care in rural settings: A qualitative descriptive study using the Theoretical Domains Framework and COM-B Model
Source: Can J Pain. 2026 Feb 5;10(1):2602540. doi: 10.1080/24740527.2025.2602540 (PMC12885414; doi:10.1080/24740527.2025.2602540)
Supplement: Supplemental Material [file UCJP_A_2602540_SM0582.docx]

| SUPPLEMENTAL TABLE 1. Theoretical Domains Framework (TDF) domains | | |
| --- | --- | --- |
| Component | **Definition** | **Component Constructs** |
| Knowledge | An awareness of the existence of something | Knowledge  Knowledge of condition Scientific rationale  Procedural knowledge  Knowledge of environment |
| Skills | An ability or proficiency acquired through practice | Skills  Skill development  Competence  Ability  Interpersonal skills  Practice  Skill assessment |
| Social/professional role identity | A coherent set of behaviours and displayed personal qualities of an individual in a social of work setting | Professional identity  Professional role  Social identity  Identity  Professional boundaries  Professional confidence  Group identity  Leadership  Organizational commitment |
| Beliefs about capabilities | Acceptance of the truth, reality of validity about an ability, talent or facility that a person can put to constructive use | Self-confidence  Perceived competence  Self-efficacy  Perceived behavioural control  Beliefs  Self-esteem  Empowerment  Professional confidence |
| Optimism | The confidence that things will happen for the best of that desired goals will be attained | Optimism  Pessimism  Unrealistic optimism  Identity |
| Beliefs about consequences | Acceptance of the truth, reality, or validity about outcomes of a behaviour in a given situation | Beliefs  Outcome expectancies  Characteristics of outcomes  Expectancies  Anticipated regret  Consequences |
| Reinforcement | Increasing the probability of a response by arranging a dependent relationship, or contingency, between the response and a given stimulus | Rewards  Incentives  Punishment  Consequences  Reinforcement  Contingencies  Sanctions |
| Intentions | A conscious decision to perform a behaviour or a resolve to act in a certain way | Stability of intentions  Stages of change model  Transtheoretical model and stages of change |
| Goals | Mental representation of outcomes or end states that an individual wants to achieve | Goals (distal/proximal)  Goal priority  Goal/target setting  Goals (autonomous/controlled)  Action planning  Implementation intention |
| Memory, attention, and decision processes | The ability to retain information, focus selectively on aspects of the environment and choose between two or more alternatives | Memory  Attention  Attention control  Decision making  Cognitive overload/tiredness |
| Environmental context and resources | Any circumstance of a person’s environment that discourages or encourages the development of skills and abilities, independence, social competence, and adaptive behaviour | Environmental stressors  Resources/material resources  Organizational culture/climate  Salient events/critical incidents  Person x environment interaction  Barriers and facilitators |
| Social influences | Those interpersonal processes that can cause individuals to change their thoughts, feelings, or behaviours | Social pressure  Social norms  Group conformity  Social comparisons  Group norms  Social support  Power  Intergroup conflict  Alienation  Group identity  Modelling |
| Emotion | A complex reaction pattern involving experiential, behavioural, and physiological elements by which the individual attempts to deal with a personally significant matter or event | Fear  Anxiety  Affect (positive/negative)  Stress  Depression  Burn out |
| Behavioural regulation | Anything aimed at managing or changing objectively observed or measured actions | Self-monitoring  Breaking habit  Action planning |
| Note. Theoretical Domains Framework domains with definitions and component constructs. Adapted from: Atkins et al., (2017). A guide to using the TDF of behaviour change to investigate implementation problems. *Implementation Science*, *12*(77), 1-18. | | |
